# IL-2 Mediates CD4^+^ T Cell Help in the Breakdown of Memory-Like CD8^+^ T Cell Tolerance under Lymphopenic Conditions

**DOI:** 10.1371/journal.pone.0012659

**Published:** 2010-09-10

**Authors:** Cécile Le Saout, Marine Villard, Clémence Cabasse, Chantal Jacquet, Naomi Taylor, Javier Hernandez

**Affiliations:** Institut de Génétique Moléculaire de Montpellier, UMR 5535 Centre National de la Recherche Scientifique, Université Montpellier 2 and Université Montpellier 1, Montpellier, France; New York University, United States of America

## Abstract

**Background:**

Lymphopenia results in the proliferation and differentiation of naïve T cells into memory-like cells in the apparent absence of antigenic stimulation. This response, at least in part due to a greater availability of cytokines, is thought to promote anti-self responses. Although potentially autoreactive memory-like CD8^+^ T cells generated in a lymphopenic environment are subject to the mechanisms of peripheral tolerance, they can induce autoimmunity in the presence of antigen-specific memory-like CD4^+^ T helper cells.

**Methodology/Principal Findings:**

Here, we studied the mechanisms underlying CD4 help under lymphopenic conditions in transgenic mice expressing a model antigen in the beta cells of the pancreas. Surprisingly, we found that the self-reactivity mediated by the cooperation of memory-like CD8^+^ and CD4^+^ T cells was not abrogated by CD40L blockade. In contrast, treatment with anti-IL-2 antibodies inhibited the onset of autoimmunity. IL-2 neutralization prevented the CD4-mediated differentiation of memory-like CD8^+^ T cells into pathogenic effectors in response to self-antigen cross-presentation. Furthermore, in the absence of helper cells, induction of IL-2 signaling by an IL-2 immune complex was sufficient to promote memory-like CD8^+^ T cell self-reactivity.

**Conclusions/Significance:**

IL-2 mediates the cooperation of memory-like CD4^+^ and CD8^+^ T cells in the breakdown of cross-tolerance, resulting in effector cytotoxic T lymphocyte differentiation and the induction of autoimmune disease.

## Introduction

CD8^+^ T cells play a key role in host defense against pathogens. The diversity of their TCR repertoire assures recognition of the vast majority of potential infectious agents. However, an important consequence of this diversity is the risk of pathogenic anti-self responses. Although the immune system has developed mechanisms of peripheral tolerance that prevent self-reactivity, CD8^+^ T cells may become activated, under conditions not yet well understood, resulting in autoimmunity. Many T cell-mediated autoimmune diseases have an extremely complex etiology with multiple genetic and environmental factors contributing to disease. This indicates that CD8^+^ T cells need to override multiple checkpoints, including the requirements for activation signals for antigen presenting cells (APCs) and CD4^+^ T helper cells as well as bypassing regulatory T cell (Treg) suppression and molecular negative T cell regulators, in order to become pathogenic effectors [1], [2]. Our increasing understanding of these control mechanisms has opened new opportunities for therapeutic interventions in autoimmunity as well as cancer immunotherapy, since the latter is generally limited by tolerance [3], [4], [5].

Lymphopenia has been linked to autoimmunity in many different murine models and more circumstantially in patients [6], [7]. Furthermore, lymphodepletion enhances anti-self-tumor antigen responses after adoptive T cell immunotherapy [8], [9]. These observations point to lymphopenia as a factor that perturbs the mechanisms of peripheral tolerance. At least three important features, common to most of the models assessed thus far, may explain how lymphopenia interferes with tolerance. First, lymphopenia may result in an imbalance between pathogenic and Tregs, with a preferential loss of the later. This is observed when pathogenic T cells are transferred into severely lymphopenic hosts and in 3-day old thymectomized mice [10], [11]. Also, chemically induced lymphopenia may induce a preferential loss of Tregs [12]. However, the loss of Tregs cannot in itself explain self-reactivity since their absence in lymphoreplete adult animals does not result in autoimmunity [13], [14]. Second, total body irradiation as well as the absence of Tregs may result in the generalized activation of APCs [15], [16]. Third, lymphopenia induces the antigen-independent activation of potentially autoreactive T cells. Naïve T cells proliferate under acute lymphopenic conditions in response to the same factors that promote their survival in lymphoreplete mice, the cytokine IL-7 and TCR engagement with self-peptide/MHC complexes [17], [18], [19], [20], [21], [22]. This proliferation is accompanied by a direct differentiation into memory-like T cells in the apparent absence of antigenic stimulation [23], [24], [25]. Indeed, these cells are functionally and phenotypically similar to *bona-fide* memory cells [26]. However, subtle differences have recently been observed in their homing and expansion capabilities [27]. Interestingly, It has been shown that memory-like T cells are less prone to tolerization than naïve cells, most likely due to their less stringent requirements for activation [28].

We have previously shown that potentially autoreactive memory-like CD8^+^ T cells are able to induce autoimmunity under lymphopenic conditions [29]. This was evaluated by transfer of transgenic Clone 4 CD8^+^ T cells bearing an inluenza virus hemagglutinin (HA)-specific, H2-K^d^ restricted TCR into lymphopenic mice wherein HA is expressed under the control of the rat insulin promoter in the beta cells of the pancreas (termed InsHA mice). However, the onset of autoimmunity was strictly dependent on the presence of HA-specific CD4^+^ T helper cells from HNT transgenic mice [29]. Similar results were obtained in the RIP-GP mouse model, where *in vitro* activated CD4^+^ T helper cells promoted the autoimmune attack of GP-specific endogenous CD8^+^ T cells under lymphopenic conditions [30]. Interestingly, in our model, help could be provided by memory-like CD4^+^ T cells generated *in vivo* upon lymphopenia-induced proliferation. These helper cells promoted the further differentiation of memory-like CD8^+^ T cells into cytotoxic T lymphocytes (CTL) in response to antigen cross-presentation and their migration to the site of antigen-expression [29]. In the absence of help, however, memory-like CD8^+^ T cells were tolerized in the draining lymph nodes (LN) of the pancreas [29]. Importantly, the cotransfer of naïve HA-specific CD8^+^ and CD4^+^ T cells into lymphoreplete InsHA mice did not result in autoimmunity and CD8^+^ T cells were tolerized under these conditions [29], [31]. Thus, overcoming cross-tolerance requires the cooperation of memory-like CD8^+^ and CD4^+^ T cells.

Many studies have assessed the helper dependent generation of primary CTL responses [32]. It is generally accepted that CD4^+^ help is required under non-inflammatory conditions where dendritic cells are not sufficiently activated by pathogen associated molecular patterns [32]. Under these conditions, CD4^+^ T helper cells would be required to activate antigen presenting dendritic cells, through the engagement of CD40, that in turn would efficiently activate naïve CD8^+^ T cells by expressing co-stimulatory receptors and cytokines [33], [34], [35]. However, there is a dearth of information on the molecular pathways involved in the cooperation between memory-like CD4^+^ and CD8^+^ T cells. In this study, we address the molecular basis of this cooperation. We found a major role for the IL-2 cytokine in the helper-dependent differentiation of memory-like Clone 4 CD8^+^ T cells into effectors capable of inducing autoimmunity and overcome cross-tolerance in lymphopenic InsHA mice.

## Results

### CD40L blockade does not prevent the induction of autoimmune diabetes by memory-like T cells

As we previously reported [29], cotransfer of naïve HA-specific Clone 4 CD8^+^ T cells together with HNT CD4^+^ T cells into sublethaly irradiated InsHA mice promoted the onset of autoimmune diabetes in the vast majority of animals between days 9 and 13 (Table 1). In this model, lymphopenia induced the proliferation and differentiation of both potentially autoreactive CD8^+^ as well as CD4^+^ T cells into memory-like cells. Upon antigen encounter, cross-presented in the draining lymph nodes of the pancreas, the further differentiation of memory-like CD8^+^ T cells into effector CTL that migrated to the pancreas and mediated beta cell destruction was dependent on antigen-specific memory-like CD4^+^ T cell help [29]. To determine the molecular basis of this cooperation, we first targeted the CD40L/CD40 pathway, thought to be the main pathway involved in the provision of CD4^+^ help [32], by using the anti-CD40L blocking monoclonal antibody (mAb) MR1 [36]. This well characterized mAb has been extensively used to block CD40 engagement by CD40L expressing cells. Lymphopenic InsHA mice, coinjected with equal numbers of Clone 4 CD8^+^ and HNT CD4^+^ T cells, were treated every other day with 0,5 mg of the MR1 mAb. Surprisingly, the vast majority of treated mice still developed autoimmune diabetes (11/12), although the onset was slightly delayed (day 13±2 as compared to day 10±1 in control mice that received an isotype control mAb).

**Table 1:** IL-2 mediates CD4 help for memory-like Clone 4 CD8^+^ T cells in the induction of autoimmune diabetes.

Cell Transfer^a^		Treatment^b^	Diabetes incidence^c^		
Clone 4 CD8^+^+ HNT CD4^+^	5×10^6^+5×10^6^	+ isotype control	100%	(n = 13)	d 10±1
		+ α-CD40L_(MR-1)_	92%	(n = 12)	d 13±2
Clone 4 CD8^+^+ HNT CD4^+^	5×10^6^+5×10^6^	+ isotype control	85%	(n = 13)	d 11±1
		+ α-IL-2_(S4B6+JES6-1)_	38%	(n = 16)	d 15±2

a Purified transgenic CD8^+^ and CD4^+^ T cells were injected as indicated into irradiated InsHA mice, 24 h after irradiation.

b Mice were treated either with MR1 anti-CD40L mAb or S4B6+JES6-1 anti-IL-2 mAbs or with their respective isotype matched antibodies.

c The onset of autoimmunity was evaluated by measuring blood glucose levels. Mice were followed over a 21 day period and were considered diabetic when levels were above 300 mg/dl in two consecutive measurements. The day of disease onset (d) is indicated.

**Figure 1:**
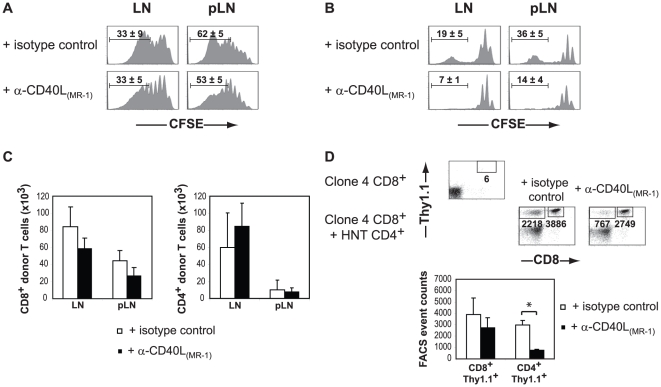
Analysis of proliferating donor T cells in anti-CD40L treated InsHA mice. Irradiated InsHA mice were injected with either CFSE-labeled naïve Clone 4 Thy1.1^+^ CD8^+^ T cells or with a combination of CFSE-labeled Clone 4 Thy1.1^+^ CD8^+^ T cells and CFSE-labeled HNT Thy1.1^+^ CD4^+^ T cells. Mice were treated with either an anti-CD40L mAb (MR1) or an isotype matched control antibody and the presence of donor T cells in different organs was analyzed on day 13 after transfer. Data from one representative experiment out of four, including an average of 3 mice per group, are presented. Levels of CFSE fluorescence on gated CD8^+^ (**A**) or CD8^−^ (**B**) Thy1.1^+^ donor lymphocytes in LN and pLN are shown. The mean percentages (± SD, *n* = 3) of highly proliferating (≥5 rounds of division) CD8^+^ or CD4^+^ donor T cells, respectively, are indicated. (**C**) Absolute numbers of donor T cells in the lymphoid organs were enumerated following treatment with with MR1 anti-CD40L mAb (black bars) and isotype control antibody (empty bars). Results are presented as means ± SD (*n* = 4). (**D**) The presence of CD8^+^ Thy1.1^+^ and CD8^−^ Thy1.1^+^ donor T cells in the pancreas was evaluated by FACS. Numbers indicate FACS event counts in the depicted gates. Graphs represent mean (± SD, *n* = 3) recovered CD8^+^ or CD4^+^ donor T cells in groups of mice described in panel C.

Analyses of the CFSE profiles of donor CD8^+^ T cells showed extensive proliferation in draining and non draining LN for both groups, although the proliferation rate was slightly delayed in the pancreatic (p)LN of anti-CD40 treated mice (Fig. 1A). However, neither absolute numbers of recovered Clone 4 CD8^+^ T cells nor migration of donor CD8^+^ T cells to the pancreas differed significantly in anti-CD40 as compared to isotype treated mice (Fig. 1C and D).

**Figure 2:**
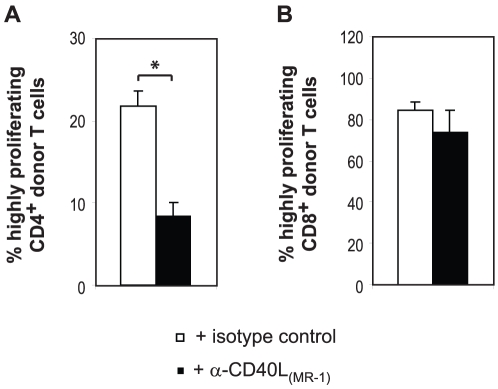
Anti-CD40L treatment inhibits rapid lymphopenia-induced CD4^+^ T cell proliferation. Irradiated BALB/c mice were injected with CFSE-labeled naïve Clone 4 Thy1.1^+^ CD8^+^ T cells and CFSE-labeled HNT Thy1.1^+^ CD4^+^ T cells. Mice were treated with either an anti-CD40L mAb (MR1) or an isotype matched antibody and the presence of donor T cells in LN was analyzed on day 13 after transfer. Levels of CFSE fluorescence on gated Thy1.1^+^ donor lymphocytes were analyzed. The mean percentages (± SD, *n* = 3) of highly proliferating CD4^+^ (**A**) and CD8^+^ (**B**) donor T cells are indicated.

Since the MR1 mAb targets the interaction between CD4^+^ T cells and APCs, we next analyzed its effect on transferred HNT CD4^+^ T cells. Donor CD4^+^ T cells in control mice showed a bimodal proliferation profile, with most cells having undergone one or two rounds of division and some cells having undergone five or more rounds (Fig. 1B). This behavior has previously been observed for adoptively transferred polyclonal CD4^+^ T cells into irradiated mice [37]. Interestingly, anti-CD40L treatment almost completely reduced the presence of highly proliferating CD4^+^ T cells in draining but, also, in non-draining LN (Fig. 1B). Although absolute numbers of recovered CD4^+^ T cells from the LN were not significantly different, we found a highly significant three-fold reduction in the CD4^+^ T cells that migrated to the pancreas of InsHA mice (Fig. 1D). This was not surprising because only highly proliferating CD62L^lo^ T cells were detected in the pancreas of non-treated mice (data not shown). To determine whether the blocking effect of MR1 on HNT CD4^+^ T cell proliferation was driven by lymphopenia and/or the HA antigen, we performed adoptive transfer experiments in lymphopenic MR1 treated BALB/c mice. In this HA antigen-independent environment, anti-CD40L again reduced the numbers of highly proliferating CD4^+^ T cells (Fig. 2A), whereas proliferation profiles of donor CD8^+^ T cells were not significantly different (Fig. 2B). Taken together, our results show that CD40L blockade has an unsuspected effect on the lymphopenia-induced proliferation of donor CD4^+^ T cells but does not prevent their ability to promote the CD8^+^ T cell-mediated onset of disease.

### IL-2 neutralization prevents autoimmune diabetes induced by the cooperation of memory-like CD4^+^ and CD8^+^ T cells

We next targeted IL-2, a cytokine mainly produced by CD4^+^ T cells, whose role in help for CD8^+^ T cell responses remains controversial [32], [38]. To assess the effect of inhibiting a potential IL-2 mediated response in the onset of disease in InsHA mice, we utilized a combination of two neutralizing mAbs, S4B6 and JES6-1 recognizing different epitopes on the IL-2 molecule [39]. Large amounts of S4B6 have been shown to neutralize circulating IL-2 in vivo inducing a decrease in Tregs, which are dependent on IL-2 for their homeostasis ([40] and Fig. S1). However, this mAb can potentially form an immune complex with IL-2 and induce CD8^+^ T cell proliferation [39]. Thus, we used S4B6 in combination with JES6-1 because it has been shown that the later can block the effect of IL-2/S4B6 complexes in vitro [39]. Lymphopenic InsHA mice injected with Clone 4 CD8^+^ and HNT CD4^+^ T cells were treated with 0.5 mg of a 1∶1 combination of S4B6 and JES6-1 every other day. Notably, anti-IL-2 treatment prevented the onset of autoimmune diabetes in 62% of treated mice (10/16), whereas most of the mice in the isotype control mAb treated group (10/11) developed disease (Table 1). Furthermore, all but one of the anti-IL-2 treated mice that showed hyperglycemia developed a milder form of disease remaining alive until the end of the experiment. This is in marked contrast to control mice where disease developed very rapidly, becoming moribund within three days following the onset of hyperglycemia.

**Figure 3:**
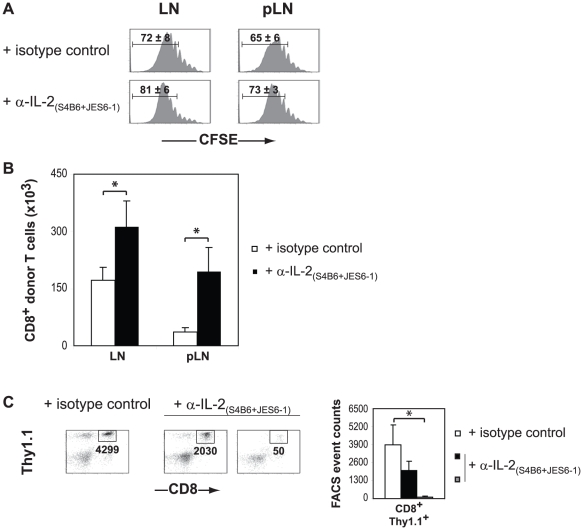
Anti-IL-2 treatment inhibits migration of Clone 4 CD8^+^ T cells to the pancreas of InsHA mice. Irradiated InsHA injected with CFSE-labeled naive Clone 4 Thy1.1^+^ CD8^+^ T cells plus HNT Thy1.1^+^ CD4^+^ T cells were treated with either a combination of anti-IL-2 mAbs (S4B6+ JES6-1) or an isotype matched control antibody. Mice were sacrificed on day 12 after transfer and the presence of donor T cells in different organs was analyzed. Data from one representative experiment out of four, including an average of 3 mice per group, are presented. (**A**) Levels of CFSE fluorescence on gated CD8^+^ Thy1.1^+^ donor lymphocytes in LN and pLN are shown. The mean percentages (± SD, *n* = 3) of highly proliferating CD8^+^ donor T cells are indicated. (**B**) Absolute numbers of CD8^+^ donor T cells in the lymphoid organs were enumerated following the treatment with anti-IL-2 mAbs (S4B6+ JES6-1) (black bars) and an isotype matched control antibody (empty bars). Results are presented as means ± SD (*n* = 3). (**C**) The presence of CD8^+^ Thy1.1^+^ donor T cells in the pancreas was evaluated by FACS. Numbers indicate FACS event counts in the depicted gates. Graphs represent mean (± SD, *n* = 3) recovered CD8^+^ donor T cells in control mice (empty bars), anti-IL-2 treated mice containing >10^3^ donor cells (black bars) and anti-IL-2 treated mice containing <10^3^ donor cells (grey bars).

In agreement with these results, we found that IL-2 neutralization blocked the migration of donor CD8^+^ T cells into the pancreas in 50% of mice analyzed at day 13 post transfer and reduced the number of infiltrating cells in the remaining mice (Fig. 3C). We found no significant differences in the proliferation profiles of donor CD8^+^ T and CD4^+^ T cells from treated and non-treated animals in either draining or non-draining lymph nodes where proliferation was extensive (Fig. 3A and data not shown). Surprisingly, anti-IL-2 treatment enhanced the accumulation of Clone 4 CD8^+^ T cell in the lymphoid tissue (Fig. 3B). Collectively, our data suggest that IL-2 plays a major role in the induction of autoimmune diabetes by memory-like Clone 4 CD8^+^ T cells.

### IL-2 neutralization blocks the CD4-mediated differentiation of memory-like CD8^+^ T cells into effectors in response to self-antigen cross-presentation

In the experiments described above, most of the CD8^+^ T cell proliferation observed in lymphoid organs was driven by the state of lymphopenia itself masking the potential proliferation induced by the cross-presentation of low amounts of the HA self-antigen in the pLN. It is precisely in this location where CD4^+^ T helpers exert their effects by enhancing the proliferation of memory-like CD8^+^ T cells in response to antigen cross-presentation and promoting their differentiation into effectors [29]. To specifically investigate this process, we performed double transfer experiments in which homeostatic proliferation/differentiation was uncoupled from antigen recognition. InsHA mice were irradiated and injected with naïve HNT CD4^+^ T cells. After 12 days, all mice were injected with lymphocytes from naïve non-irradiated InsHA mice in order to “fill” the space and prevent any subsequent lymphopenia-induced proliferation. After resting for an additional 4 days, InsHA mice received CFSE labeled memory-like Clone 4 CD8^+^ T cells that had been generated by their prior transfer into irradiated BALB/c mice. At the time of secondary transfer, Clone 4 cells isolated from irradiated BALB/c mice were CD44^hi^ CD25^−^ CD62L^hi^, a typical central memory phenotype. Five days later, InsHA mice were sacrificed and proliferation of donor Clone 4 cells was assessed in the lymphoid organs.

**Figure 4:**
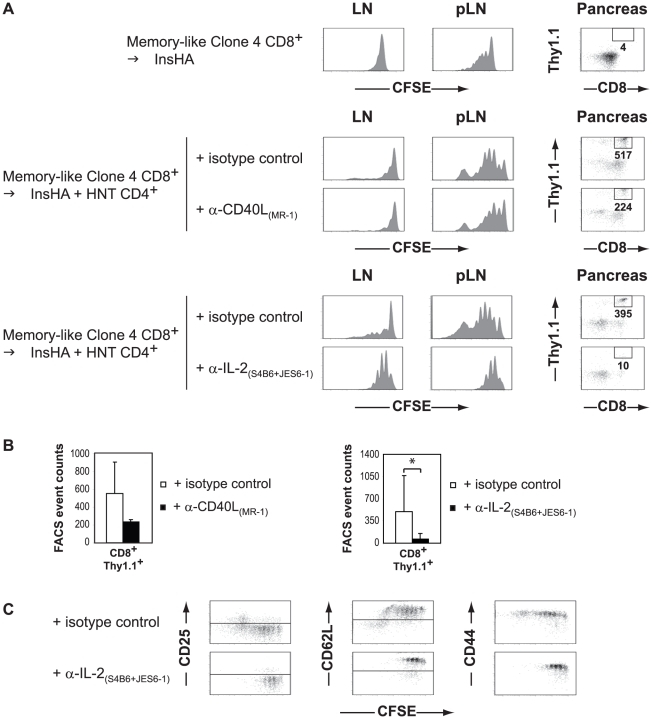
Anti-IL-2 treatment prevents the differentiation of memory-like Clone 4 CD8^+^ T cells into effectors in response to self-antigen cross-presentation. Irradiated InsHA mice were either injected with 5×10^6^ CFSE-labeled naïve HNT Thy1.1^+^ CD4^+^ T cells or PBS. On day 12, both groups of mice were “refilled” with 100×10^6^ spleen and LN cells from unmanipulated InsHA mice. Four days later, the “refilled” InsHA mice received enriched CFSE-labeled memory-like Clone 4 Thy1.1^+^ CD8^+^ T cells, generated by injection of naïve cells into irradiated BALB/c mice 16 days previously. Mice adoptively transferred with HNT CD4^+^ T cells were also treated with either anti-CD40L, anti-IL-2 or the respective isotype matched control antibodies. Five days after secondary transfer, InsHA mice were sacrificed and donor CD8^+^ Thy1.1^+^ lymphocytes from pancreas, LN and pLN were analyzed by FACS. Data from one representative experiment of four are presented. (A) Histograms represent CFSE fluorescence on gated CD8^+^ Thy1.1^+^ lymphocytes. The presence of CD8^+^ Thy1.1^+^ donor T cells in the pancreas was evaluated by FACS. FACS event counts in the depicted gates are indicated. (B) Graphs represent mean (± SD, *n* = 3–8 per group) recovered CD8^+^ donor T cells from the pancreas of mice from indicated groups. (C) Expression of CD25, CD62L and CD44 on gated CD8^+^ Thy1.1^+^ lymphocytes of mice treated with anti-IL-2 mAbs was assessed by flow cytometry. Plots represent expression of CD25, CD62L and CD44 as a function of CFSE fluorescence on gated CD8^+^ Thy1.1^+^ lymphocytes in the pLN.

As we have previously shown, HNT CD4^+^ T cells dramatically enhanced the proliferation of memory-like Clone 4 CD8^+^ T cells in response to self-antigen cross-presentation in the pLN and, more importantly, it was only in their presence that the Clone 4 CD8^+^ T cells migrated to the pancreas ([29] and Fig. 4A). Treatment with anti-CD40L did not significantly modify the proliferation profiles of Clone 4 CD8^+^ T cells (Fig. 4A). In marked contrast, IL-2 neutralization prevented the enhanced proliferation induced by CD4 help in the pLN of InsHA mice (Fig. 4A). Notably, IL-2 neutralization blocked the migration of CD8^+^ T cells to the pancreas in all animals tested, whereas anti-CD40L only had a moderate effect (Fig. 4A and B).

Finally, we assessed the phenotype of proliferating Clone 4 CD8+ T cells in the pLN. As previously described, CD4 help induced the differentiation of highly proliferating memory-like CD8^+^ T cells into effectors as demonstrated by the dowregulation of CD62L and the upregulation of CD25 in control mice (Fig. 4C) [29]. IL-2 neutralization also blocked this differentiation (Fig. 4C), whereas anti-CD40L did not (data not shown). It is important to note that while anti-IL-2 treatment prevented antigen driven proliferation and differentiation in the pLN, it also induced a non-specific proliferation of memory-like CD8^+^ T cells in non-draining LN (Fig. 4A). Proliferating cells retained a central memory phenotype (Fig. 4C). This may reflect the formation of an immune complex between S4B6 mAb and endogenous IL-2 [39] and the inability of JES6-1 mAb to completely prevent their immunostimulatory effects on memory-like CD8^+^ T cells *in vivo* and would explain the enhanced accumulation of Clone 4 cells in the LN of long term anti-IL-2 treated mice that did not develop diabetes (see Fig. 3B).

### IL-2 signaling promotes memory-like CD8^+^ T cell mediated autoimmune diabetes in the absence of CD4^+^ help

**Figure 5:**
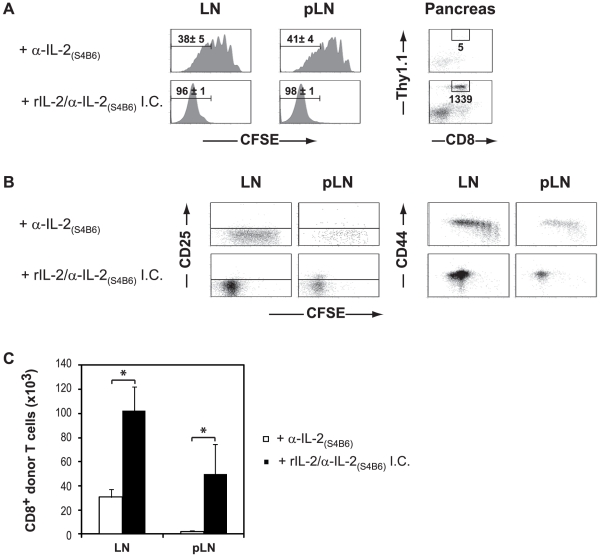
IL-2 immune complex induces the differentiation of memory-like Clone 4 CD8^+^ T cells into effectors and their migration to the pancreas. Irradiated InsHA mice injected with 5×10^6^ CFSE-labeled naïve Clone 4 Thy1.1^+^ CD8^+^ T cells were treated with IL-2/S4B6 anti-IL-2 immune complex (I.C.) or anti-IL-2. Mice were sacrificed on day 11 after transfer. Data from one representative experiment of three are presented. (A) Levels of CFSE fluorescence on gated CD8^+^ Thy1.1^+^ donor lymphocytes in LN and pLN are shown. The mean percentages (± SD, *n* = 3) of proliferating CD8^+^ donor T cells are indicated. The presence of CD8^+^ Thy1.1^+^ donor T cells in the pancreas was evaluated by FACS. FACS event counts in the depicted gates are indicated. (B) Plots represent expression of CD25 and CD44 as a function of CFSE fluorescence on gated CD8^+^ Thy1.1^+^ lymphocytes in the pLN. (C) Absolute numbers of CD8^+^ donor T cells in the lymphoid organs were enumerated following treatment with with IL-2/S4B6 I.C. (black bars) and S4B6 mAb (empty bars). Results are presented as means ± SD (*n* = 3).

To determine whether the induction of an IL-2 signaling cascade in CD8^+^ T cells was sufficient to account for the effects of CD4 help in the onset of autoimmune diabetes in InsHA mice, we sought to substitute HNT CD4^+^ T cells by an exogenous source of IL-2. However, daily treatment of lymphopenic InsHA mice for 5 consecutive days with recombinant IL-2 did not result in the onset of disease following transfer of Clone 4 CD8^+^ T cells (data not shown). We hypothesized that the absence of an IL-2 mediated effect might have been due to its extremely short half-life *in vivo*. It has recently been shown that the *in vitro* formation of IL-2/anti-IL-2 mAb immune complex prior to injection can augment the half-life and functionality of IL-2 [39], [41], [42]. Therefore, we next utilized IL-2/S4B6 (2 µg/10 µg) immune complex as an exogenous source of IL-2. Notably, 100% of the IL-2/S4B6 treated InsHA mice developed autoimmune diabetes following adoptive transfer of Clone 4 CD8^+^ T cells alone (Table 2). This was associated with a dramatically enhanced proliferation of Clone 4 CD8^+^ T cells in the pLN as well as in non draining LN (Fig. 5A and C). This is in agreement with previous observations showing that this immune complex induces antigen-independent expansion of memory phenotype CD8^+^ T cells [39]. Most importantly, IL-2 complexes promoted the differentiation of memory-like Clone 4 CD8^+^ T cells into effectors capable of migrating to the pancreas, only in the pLN (Fig. 5B). In marked contrast, control mice treated with this low dose of S4B6 mAb alone did not develop hyperglycemia and remained healthy over the course of the experiments (Table 2). Thus, IL-2 in the form of an immune complex can efficiently substitute for CD4 help, promoting the differentiation of CTL capable of overcoming self-tolerance and inducing autoimmunity under lymphopenic conditions.

**Table 2:** IL-2 immune complex promote memory-like Clone 4 CD8^+^ T cell-mediated autoimmune diabetes in the absence of CD4 help.

Cell Transfer^a^		Treatment^b^	Diabetes incidence^c^		
Clone 4 CD8^+^	5×10^6^	+ α-IL-2_(S4B6)_	0%	(n = 7)	
		+ rIL-2/α-IL-2_(S4B6)_ I.C.	100%	(n = 9)	d 11±1

a Purified transgenic CD8^+^ T cells were injected as indicated into InsHA mice 24 h after irradiation.

b Mice were treated with IL-2/S4B6 immune complex (I.C.) or S4B6 alone.

c Monitoring of lymphopenic InsHA mice was performed as described in Table 1.

## Discussion

In this report, we examined the mechanisms via which memory-like CD8^+^ and CD4^+^ T cells cooperate to overcome cross-tolerance and induce self-reactivity under lymphopenic conditions. Our findings demonstrate a key role for IL-2 in the CD4-mediated differentiation of memory-like CD8^+^ T cells into diabetogenic effectors. We showed that IL-2 neutralization prevents the differentiation of the CD8^+^ T cells in response to self-antigen cross-presentation in the pLN and their subsequent migration to the pancreas. Furthermore, in the absence of helper cells, IL-2 signaling is sufficient to transform a tolerogenic antigenic stimulus for memory-like CD8^+^ T cells into a priming stimulus [43].

IL-2 has long been known for its adjuvant effects on CD8^+^ T cell expansion. For this reason, it was proposed as the factor that CD4^+^ T helper cells provide for CD8^+^ T cell responses [44]. However, the finding that IL-2 deficient mice are able to mount primary CD8^+^ T cell responses and that CD4^+^ T cells condition DC, through CD40L/CD40 interactions, to efficiently prime CD8^+^ T cells disfavored the original hypothesis [33], [34], [35], [45]. Recently though, it has been shown that IL-2 is required for the generation of functional anti-pathogen memory CD8^+^ T cells [46], [47]; IL-2 appears to be needed at the time of priming and can be provided by antigen-specific CD4^+^ T helper cells [47], [48]. Thus, continued IL-2 signaling in CD8^+^ T cells promotes their differentiation into short-lived effectors [49], [50], [51]. Nonetheless, in certain studies, IL-2 was not essential for the differentiation of CD8^+^ T cells into effectors during the primary response [46], [47]. IL-2 may be dispensable in this latter context where CD8^+^ T cells would benefit from inflammatory signals during an infection with the secretion of other factors with partly overlapping functions, such as type I interferons and IL-12 [52], [53], [54] [51]. In our model, where antigen encounter is likely to occur in the absence of overt inflammation (in the absence of help, Clone 4 cells are still tolerized in irradiated mice and do not differentiate into effectors [29]), IL-2 appears to be the major factor driving help-dependent differentiation of memory-like CD8^+^ T cells into effectors in response to self-antigen cross-presentation.

Our results show that there is no major role for the CD40L pathway in help provided by self-antigen specific HNT CD4^+^ T cells in lymphopenic InsHA mice. Previous reports have shown that CD4 help could be fully or partially independent of the CD40L/CD40 pathway [31], [55], [56], but our finding was somewhat surprising in light of a recent study showing that CD8^+^ T cells proliferating under lymphopenic conditions required CD4 help to induce autoimmunity and that this help was mediated by CD40 engagement on APCs [30]. However, in this previous study, help was provided by CD4^+^ T cells activated *in vitro* with antigen whereas, in the experiments reported here help was provided by memory-like CD4^+^ T cells generated *in vivo* upon lymphopenia induced proliferation. Memory phenotype CD4^+^ T cells and effector cells express very different levels of CD40L ([48], [57] and data not shown). It is interesting to speculate that the differentiation state of the CD4^+^ T helper cells could regulate the pathway utilized to provide help. Furthermore, it has been shown that irradiation promotes the upregulation of co-stimulatory molecules on dendritic cells by mean of the release of TLR ligands [15]. Since lymphopenia is induced by irradiation in our model, activation of the cross-presenting APCs could by-pass the need for CD40 engagement by CD4^+^ T helpers.

Notably, we found an unsuspected role for CD40L in the lymphopenia-induced proliferation of CD4^+^ T cells. This is in apparent contradiction with a previous study showing that CD40L co-stimulation did not play a role in lymphopenia-induced proliferation of CD4^+^ T cells in irradiated hosts [58]. However, in this report only cells undergoing one or two rounds of division, the classical homeostatic proliferation, were analyzed [58]. Our results demonstrate that the proliferation of a small proportion of cells undergoing many rounds of division in irradiated hosts is CD40L dependent.

The data presented here clearly manifest the dual role of IL-2 in maintaining self-tolerance and promoting CD8^+^ T cell immunity. Our results are consistent with recent reports showing that IL-2 not only promotes proliferation and survival of CD8^+^ T cells but also their differentiation into effectors [43], [59], [60]. Thus, IL-2 allows Clone 4 CD8^+^ T cells to overcome cross-tolerance and initiate an autoimmune response. On the other hand, Tregs are dependent on IL-2 for their growth and survival and can be eliminated by the use of neutralizing anti-IL-2 mAbs [40], [61], [62]. In our model, autoimmunity ensues while mice are lymphopenic and there is a likely imbalance between effector cells and Tregs [29]. The use of neutralizing anti-IL-2 mAbs in InsHA mice not only prevents the help mediated differentiation of memory-like CD8^+^ T cells but, at the same time, it further decreases the number of Tregs. One possibility as to why IL-2 neutralization was not 100% effective in preventing disease (reaching 62%, see Table 1) could be due to the additional depletion of Tregs. This observation illustrates the key role of IL-2 in regulating the fine equilibrium between CD8^+^ T cell tolerance and immunity [63], [64]. Our results, showing that IL-2 immune complex can promote the breakdown of peripheral tolerance by memory-like CD8^+^ T cells in the absence of CD4^+^ T cells help, have important implications for the development of therapies for autoimmune diseases as well as cancer immunotherapies.

## Materials and Methods

### Ethics statement

All experimental procedures were approved by the “Direction Départamentale des Services Véterinaires”, authorization number D-34-172-16 and 34.347. Blood withdrawal from the retro orbital sinus was performed under isoflurane anesthesia. Diabetic mice were sacrificed to prevent suffering by prolonged hyperglycemia.

### Mice

BALB/c mice were purchased from Charles River and then housed at the Institut de Génétique Moléculaire de Montpellier (IGMM). InsHA [65], Clone 4 TCR [66] and HNT TCR [67] (kindly provided by Linda A. Sherman, The Scripps Research Institute) transgenic mice lines were backcrossed with BALB/c mice for at least 10 generations. Clone 4 and HNT mice were then crossed with BALB/c Thy1.1^+/+^ for two generations to achieve homozygosity for Thy1.1. All mice used in these studies were between 8 and 16 weeks of age. Mice were propagated and maintained under specific pathogen-free conditions in the IGMM animal facility.

### Preparation of CFSE labeled naïve TCR transgenic T cells

CD8**^+^** T cells from Clone 4 TCR Thy1.1 transgenic mice and CD4^+^ T cells from HNT Thy1.1 were prepared from single cell LN suspensions by magnetic depletion using the T cell isolation kits and an AutoMACS apparatus from Miltenyi according to the manufacturer's instructions. T cell purity was greater than 90%. Purified T cells (2×10^7^ cells/ml) were labeled with 5 µM 5- and 6-carboxy-fluorescein succinimidyl ester (CFSE) (Molecular Probes, Eugene, OR) in PBS for 10 min at 37°C.

### Induction of lymphopenia by irradiation

BALB/c or InsHA mice were sublethaly irradiated (450 rads) utilizing a therapeutic irradiator, Variant. Under these conditions, depletion of host T cells was approximately 90% at 24 h after irradiation and 70% at 7 days. Mice were used for adoptive transfer experiments 24 h after irradiation, unless otherwise noted.

### Adoptive transfer

4–5×10^6^ CFSE-labeled Clone 4 TCR Thy1.1 CD8^+^ T cells and/or 4–5×10^6^ CFSE-labeled HNT TCR Thy1.1 CD4^+^ T cells in PBS were transferred by i.v. injection into irradiated InsHA and BALB/c mice.

For double transfer experiments, InsHA mice were irradiated and 24 h later, they either received HNT TCR Thy1.1 CD4^+^ T cells or PBS. All mice were “refilled” with a mixture of 100×10^6^ spleen and lymph node cells from non-irradiated InsHA mice 12 days later. At day 16, the InsHA mice received secondary i.v. transfers of CFSE-labeled, enriched memory-like Clone 4 TCR Thy1.1 CD8^+^ T cells. These latter cells were obtained from the lymph nodes and spleen of irradiated BALB/c mice that had been injected 16 days earlier with naive Clone 4 TCR Thy1.1 CD8^+^ T cells. Cells recovered from one single primary host were transferred into one secondary recipient mouse.

### *In vivo* treatments with antibodies and cytokines

Purified anti-CD40L (clone MR1, Hamster IgG (BioXcell): 0.5 mg/mouse suspended in PBS) was administered by i.p. injection every other day starting on day 1 after transfer until mice were hyperglycemic. In double transfer experiments, mice were treated from day 11 before CD8^+^ T cell transfer until the end of the experiment. Anti-IL-2 (Clones S4B6 and JES6-1, Rat IgG2a, (BioXcell): 0.25 mg of each mAb/mouse) was administered by i.p. injection every other day starting on day 1 after transfer. In double transfer experiments, mice were treated from the day of CD8^+^ T cell transfer until the end of the experiment. Recombinant mIL-2 was purchased from Peprotech. IL-2 immune complex, preformed in vitro by mixing 2 µg of recombinant mIL-2 and 10 µg of the S4B6 anti-IL-2 mAb in PBS, was administered by i.p. injection. Mice were treated on five consecutive days starting at day 5 after transfer.

### Diabetes monitoring

Mice were monitored for diabetes by measuring blood glucose every 4 days for a maximum period of 21 days with a Glucomatic® ESPRIT Apparatus (Bayer, France). In some instances, daily measurements were performed on urine samples with Multistix® 8 SG strips (Bayer, France). Animals were considered diabetic when glucose levels were above 300 mg/dl during two consecutive measurements.

### Flow cytometry

Pancreas, pancreatic (pLN) and a mixture of inguinal, axillary, cervical, mandibular, popliteal and mesenteric LN were excised and processed separately to obtain single cell suspensions by mechanical disruption on Nitex filters in PBS containing 5% FCS at 4°C. After counting, all pancreatic LN cells and an equivalent number of cells from other LN and spleen were stained with the indicated Abs. For the pancreas, suspensions were subject to Histopaque separation and all cells obtained from a single pancreas were stained.

All mAbs and secondary reagents were purchased from BD PharMingen (San Diego CA) except anti-CD44-APC-Alexa Fluor® 750 and anti-Foxp3-APC mAbs (eBioscience, San Diego, CA). Donor Clone 4 and HNT T cells were detected and enumerated by virtue of Thy1.1 expression. Lymphocytes were incubated with anti-CD8α-PerCP and anti-Thy1.1-PE mAbs in PBS containing 2% FCS and 0.02% sodium azide for 30 min at 4°C. After washing, cells were analyzed on a FACSCanto II apparatus using Diva software (BDB, Mountain View, CA).

For Clone 4 CD8^+^ T cells, the intensity of CFSE fluorescence was analyzed in the CD8^+^ Thy1.1^+^ subpopulation of lymphocytes. For HNT cells, this was performed by gating on CD8^−^ Thy1.1^+^ or CD4^+^ Thy1.1^+^ T cells depending on the experiment. To assess the phenotype of proliferating T cells, they were stained with anti-CD25-PE-Cy7, anti-CD62L-APC and anti-CD44-APC-Alexa Fluor® 750 mAbs. Intracellular Foxp3 staining was performed utilizing the Fixation and Permeabilization Kit (eBioscience) according to manufacturer's instructions.

### Statistical analyses

Statistical significance was determined using a Student's *t* test with a one-tailed distribution and two-sample equal variance. Data were considered to be statistically different (*) for *P* <0.05.

## Supporting Information

Figure S1 IL-2 neutralization depletes Tregs in lymphopenic InsHA mice. Mice described in Figure 3 were sacrificed at day 13 after transfer and expression of CD25, and Foxp3 was assessed on gated endogenous CD4^+^ Thy1.1^−^ T cells in LN. The mean percentages (± SD, n = 3) of CD4^+^ CD25^+^ FoxP3^+^ T cells are indicated. Data from one representative experiment out of four, including an average of three mice per group, are presented. A 30% reduction in the frequency of Tregs was observed in InsHA mice treated with anti-IL-2 mAbs as compared to isotype control treated mice.
(0.70 MB EPS)

## References

[1] Ohashi PS (2002). T-cell signalling and autoimmunity: molecular mechanisms of disease.. Nat Rev Immunol.

[2] Redmond WL, Sherman LA (2005). Peripheral tolerance of CD8 T lymphocytes.. Immunity.

[3] Guilloux Y, Viret C, Gervois N, Le Drean E, Pandolfino MC (1994). Defective lymphokine production by most CD8+ and CD4+ tumor-specific T cell clones derived from human melanoma-infiltrating lymphocytes in response to autologous tumor cells in vitro.. Eur J Immunol.

[4] Hernandez J, Lee PP, Davis MM, Sherman LA (2000). The use of HLA A2.1/p53 peptide tetramers to visualize the impact of self tolerance on the TCR repertoire.. J Immunol.

[5] Staveley-O'Carroll K, Sotomayor E, Montgomery J, Borrello I, Hwang L (1998). Induction of antigen-specific T cell anergy: An early event in the course of tumor progression.. Proc Natl Acad Sci U S A.

[6] Gleeson PA, Toh BH, van Driel IR (1996). Organ-specific autoimmunity induced by lymphopenia.. Immunol Rev.

[7] Khoruts A, Fraser JM (2005). A causal link between lymphopenia and autoimmunity.. Immunol Lett.

[8] Dudley ME, Wunderlich JR, Robbins PF, Yang JC, Hwu P (2002). Cancer regression and autoimmunity in patients after clonal repopulation with antitumor lymphocytes.. Science.

[9] Dummer W, Niethammer AG, Baccala R, Lawson BR, Wagner N (2002). T cell homeostatic proliferation elicits effective antitumor autoimmunity.. J Clin Invest.

[10] Read S, Malmstrom V, Powrie F (2000). Cytotoxic T lymphocyte-associated antigen 4 plays an essential role in the function of CD25(+)CD4(+) regulatory cells that control intestinal inflammation.. J Exp Med.

[11] Sakaguchi S (2005). Naturally arising Foxp3-expressing CD25+CD4+ regulatory T cells in immunological tolerance to self and non-self.. Nat Immunol.

[12] Brode S, Raine T, Zaccone P, Cooke A (2006). Cyclophosphamide-induced type-1 diabetes in the NOD mouse is associated with a reduction of CD4+CD25+Foxp3+ regulatory T cells.. J Immunol.

[13] Lahl K, Loddenkemper C, Drouin C, Freyer J, Arnason J (2007). Selective depletion of Foxp3+ regulatory T cells induces a scurfy-like disease.. J Exp Med.

[14] McHugh RS, Shevach EM (2002). Cutting edge: depletion of CD4+CD25+ regulatory T cells is necessary, but not sufficient, for induction of organ-specific autoimmune disease.. J Immunol.

[15] Paulos CM, Wrzesinski C, Kaiser A, Hinrichs CS, Chieppa M (2007). Microbial translocation augments the function of adoptively transferred self/tumor-specific CD8+ T cells via TLR4 signaling.. J Clin Invest.

[16] Schildknecht A, Brauer S, Brenner C, Lahl K, Schild H (2010). FoxP3+ regulatory T cells essentially contribute to peripheral CD8+ T-cell tolerance induced by steady-state dendritic cells.. Proc Natl Acad Sci U S A.

[17] Ernst B, Lee DS, Chang JM, Sprent J, Surh CD (1999). The peptide ligands mediating positive selection in the thymus control T cell survival and homeostatic proliferation in the periphery.. Immunity.

[18] Goldrath AW, Bevan MJ (1999). Low-affinity ligands for the TCR drive proliferation of mature CD8+ T cells in lymphopenic hosts.. Immunity.

[19] Kieper WC, Jameson SC (1999). Homeostatic expansion and phenotypic conversion of naive T cells in response to self peptide/MHC ligands.. Proc Natl Acad Sci U S A.

[20] Schluns KS, Kieper WC, Jameson SC, Lefrancois L (2000). Interleukin-7 mediates the homeostasis of naive and memory CD8 T cells in vivo.. Nat Immunol.

[21] Tan JT, Dudl E, LeRoy E, Murray R, Sprent J (2001). IL-7 is critical for homeostatic proliferation and survival of naive T cells.. Proc Natl Acad Sci U S A.

[22] Viret C, Wong FS, Janeway CA (1999). Designing and maintaining the mature TCR repertoire: the continuum of self-peptide:self-MHC complex recognition.. Immunity.

[23] Cho BK, Rao VP, Ge Q, Eisen HN, Chen J (2000). Homeostasis-stimulated proliferation drives naive T cells to differentiate directly into memory T cells.. J Exp Med.

[24] Goldrath AW, Bogatzki LY, Bevan MJ (2000). Naive T cells transiently acquire a memory-like phenotype during homeostasis-driven proliferation.. J Exp Med.

[25] Murali-Krishna K, Ahmed R (2000). Cutting edge: naive T cells masquerading as memory cells.. J Immunol.

[26] Hamilton SE, Wolkers MC, Schoenberger SP, Jameson SC (2006). The generation of protective memory-like CD8+ T cells during homeostatic proliferation requires CD4+ T cells.. Nat Immunol.

[27] Cheung KP, Yang E, Goldrath AW (2009). Memory-like CD8+ T cells generated during homeostatic proliferation defer to antigen-experienced memory cells.. J Immunol.

[28] Wu Z, Bensinger SJ, Zhang J, Chen C, Yuan X (2004). Homeostatic proliferation is a barrier to transplantation tolerance.. Nat Med.

[29] Le Saout C, Mennechet S, Taylor N, Hernandez J (2008). Memory-like CD8+ and CD4+ T cells cooperate to break peripheral tolerance under lymphopenic conditions.. Proc Natl Acad Sci U S A.

[30] Calzascia T, Pellegrini M, Lin A, Garza KM, Elford AR (2008). CD4 T cells, lymphopenia, and IL-7 in a multistep pathway to autoimmunity.. Proc Natl Acad Sci U S A.

[31] Hernandez J, Aung S, Marquardt K, Sherman LA (2002). Uncoupling of proliferative potential and gain of effector function by CD8(+) T cells responding to self-antigens.. J Exp Med.

[32] Castellino F, Germain RN (2006). Cooperation between CD4+ and CD8+ T cells: when, where, and how.. Annu Rev Immunol.

[33] Bennett SR, Carbone FR, Karamalis F, Flavell RA, Miller JF (1998). Help for cytotoxic-T-cell responses is mediated by CD40 signalling.. Nature.

[34] Ridge JP, Di Rosa F, Matzinger P (1998). A conditioned dendritic cell can be a temporal bridge between a CD4+ T-helper and a T-killer cell.. Nature.

[35] Schoenberger SP, Toes RE, van der Voort EI, Offringa R, Melief CJ (1998). T-cell help for cytotoxic T lymphocytes is mediated by CD40-CD40L interactions.. Nature.

[36] Noelle RJ, Roy M, Shepherd DM, Stamenkovic I, Ledbetter JA (1992). A 39-kDa protein on activated helper T cells binds CD40 and transduces the signal for cognate activation of B cells.. Proc Natl Acad Sci U S A.

[37] Kieper WC, Troy A, Burghardt JT, Ramsey C, Lee JY (2005). Recent immune status determines the source of antigens that drive homeostatic T cell expansion.. J Immunol.

[38] Malek TR, Bayer AL (2004). Tolerance, not immunity, crucially depends on IL-2.. Nat Rev Immunol.

[39] Boyman O, Kovar M, Rubinstein MP, Surh CD, Sprent J (2006). Selective stimulation of T cell subsets with antibody-cytokine immune complexes.. Science.

[40] Setoguchi R, Hori S, Takahashi T, Sakaguchi S (2005). Homeostatic maintenance of natural Foxp3(+) CD25(+) CD4(+) regulatory T cells by interleukin (IL)-2 and induction of autoimmune disease by IL-2 neutralization.. J Exp Med.

[41] Kamimura D, Sawa Y, Sato M, Agung E, Hirano T (2006). IL-2 in vivo activities and antitumor efficacy enhanced by an anti-IL-2 mAb.. J Immunol.

[42] Phelan JD, Orekov T, Finkelman FD (2008). Cutting edge: mechanism of enhancement of in vivo cytokine effects by anti-cytokine monoclonal antibodies.. J Immunol.

[43] Waithman J, Gebhardt T, Davey GM, Heath WR, Carbone FR (2008). Cutting edge: Enhanced IL-2 signaling can convert self-specific T cell response from tolerance to autoimmunity.. J Immunol.

[44] Keene JA, Forman J (1982). Helper activity is required for the in vivo generation of cytotoxic T lymphocytes.. J Exp Med.

[45] Kundig TM, Schorle H, Bachmann MF, Hengartner H, Zinkernagel RM (1993). Immune responses in interleukin-2-deficient mice.. Science.

[46] Bachmann MF, Wolint P, Walton S, Schwarz K, Oxenius A (2007). Differential role of IL-2R signaling for CD8+ T cell responses in acute and chronic viral infections.. Eur J Immunol.

[47] Williams MA, Tyznik AJ, Bevan MJ (2006). Interleukin-2 signals during priming are required for secondary expansion of CD8+ memory T cells.. Nature.

[48] Wilson EB, Livingstone AM (2008). Cutting edge: CD4+ T cell-derived IL-2 is essential for help-dependent primary CD8+ T cell responses.. J Immunol.

[49] Kalia V, Sarkar S, Subramaniam S, Haining WN, Smith KA (2010). Prolonged interleukin-2Ralpha expression on virus-specific CD8+ T cells favors terminal-effector differentiation in vivo.. Immunity.

[50] Obar JJ, Molloy MJ, Jellison ER, Stoklasek TA, Zhang W (2010). CD4+ T cell regulation of CD25 expression controls development of short-lived effector CD8+ T cells in primary and secondary responses.. Proc Natl Acad Sci U S A.

[51] Pipkin ME, Sacks JA, Cruz-Guilloty F, Lichtenheld MG, Bevan MJ (2010). Interleukin-2 and inflammation induce distinct transcriptional programs that promote the differentiation of effector cytolytic T cells.. Immunity.

[52] Ahonen CL, Doxsee CL, McGurran SM, Riter TR, Wade WF (2004). Combined TLR and CD40 triggering induces potent CD8+ T cell expansion with variable dependence on type I IFN.. J Exp Med.

[53] Le Bon A, Durand V, Kamphuis E, Thompson C, Bulfone-Paus S (2006). Direct stimulation of T cells by type I IFN enhances the CD8+ T cell response during cross-priming.. J Immunol.

[54] Le Bon A, Etchart N, Rossmann C, Ashton M, Hou S (2003). Cross-priming of CD8+ T cells stimulated by virus-induced type I interferon.. Nat Immunol.

[55] Behrens GM, Li M, Davey GM, Allison J, Flavell RA (2004). Helper requirements for generation of effector CTL to islet beta cell antigens.. J Immunol.

[56] Lu Z, Yuan L, Zhou X, Sotomayor E, Levitsky HI (2000). CD40-independent pathways of T cell help for priming of CD8(+) cytotoxic T lymphocytes.. J Exp Med.

[57] Hamilton SE, Jameson SC (2008). The nature of the lymphopenic environment dictates protective function of homeostatic-memory CD8+ T cells.. Proc Natl Acad Sci U S A.

[58] Prlic M, Blazar BR, Khoruts A, Zell T, Jameson SC (2001). Homeostatic expansion occurs independently of costimulatory signals.. J Immunol.

[59] Janas ML, Groves P, Kienzle N, Kelso A (2005). IL-2 regulates perforin and granzyme gene expression in CD8+ T cells independently of its effects on survival and proliferation.. J Immunol.

[60] Verdeil G, Marquardt K, Surh CD, Sherman LA (2008). Adjuvants targeting innate and adaptive immunity synergize to enhance tumor immunotherapy.. Proc Natl Acad Sci U S A.

[61] D'Cruz LM, Klein L (2005). Development and function of agonist-induced CD25+Foxp3+ regulatory T cells in the absence of interleukin 2 signaling.. Nat Immunol.

[62] Fontenot JD, Rasmussen JP, Gavin MA, Rudensky AY (2005). A function for interleukin 2 in Foxp3-expressing regulatory T cells.. Nat Immunol.

[63] Knoechel B, Lohr J, Kahn E, Bluestone JA, Abbas AK (2005). Sequential development of interleukin 2-dependent effector and regulatory T cells in response to endogenous systemic antigen.. J Exp Med.

[64] Tang Q, Adams JY, Penaranda C, Melli K, Piaggio E (2008). Central role of defective interleukin-2 production in the triggering of islet autoimmune destruction.. Immunity.

[65] Lo D, Freedman J, Hesse S, Palmiter RD, Brinster RL (1992). Peripheral tolerance to an islet cell-specific hemagglutinin transgene affects both CD4+ and CD8+ T cells.. Eur J Immunol.

[66] Morgan DJ, Liblau R, Scott B, Fleck S, McDevitt HO (1996). CD8(+) T cell-mediated spontaneous diabetes in neonatal mice.. J Immunol.

[67] Scott B, Liblau R, Degermann S, Marconi LA, Ogata L (1994). A role for non-MHC genetic polymorphism in susceptibility to spontaneous autoimmunity.. Immunity.

